# Betaine supplement enhances skeletal muscle differentiation in murine myoblasts via IGF-1 signaling activation

**DOI:** 10.1186/1479-5876-11-174

**Published:** 2013-07-19

**Authors:** Pamela Senesi, Livio Luzi, Anna Montesano, Nausicaa Mazzocchi, Ileana Terruzzi

**Affiliations:** 1 Department of Biomedical Sciences for Health, University of Milan, Milan, Italy; 2 Metabolism Research Centre and Department of Endocrinology and Metabolic Diseases, San Donato Hospital and Scientific Institute, Milan, Italy; 3 Division of Metabolic and Cardiovascular Sciences Metabolism, Nutrigenomics and Cellular Differentiation Unit, San Raffaele Scientific Institute, Milan, Italy

**Keywords:** Betaine, Myoblast differentiation, Muscle tissue, Hypertrophy, IGF-1, Striated muscle cell

## Abstract

**Background:**

Betaine (BET) is a component of many foods, including spinach and wheat. It is an essential osmolyte and a source of methyl groups. Recent studies have hypothesized that BET might play a role in athletic performance. However, BET effects on skeletal muscle differentiation and hypertrophy are still poorly understood.

**Methods:**

We examined BET action on neo myotubes maturation and on differentiation process, using C2C12 murine myoblastic cells. We used RT^2^-PCR array, Western blot and immunofluorescence analysis to study the BET effects on morphological features of C2C12 and on signaling pathways involved in muscle differentiation and hypertrophy.

**Results:**

We performed a dose–response study, establishing that 10 mM BET was the dose able to stimulate morphological changes and hypertrophic process in neo myotubes. RT^2^-PCR array methodology was used to identify the expression profile of genes encoding proteins involved in IGF-1 pathway. A dose of 10 mM BET was found to promote IGF-1 receptor (IGF-1 R) expression. Western blot and immunofluorescence analysis, performed in neo myotubes, pointed out that 10 mM BET improved IGF-1 signaling, synthesis of Myosin Heavy Chain (MyHC) and neo myotubes length.

In addition, we investigated BET role on myoblasts proliferation and differentiation. During proliferation, BET did not modify C2C12 proliferative rate, but promoted myogenic induction, enhancing MyoD protein content and cellular elongation. During differentiation, BET caused an increase of muscle-specific markers and IGF-1 R protein levels.

**Conclusions:**

Our findings provide the first evidence that BET could promote muscle fibers differentiation and increase myotubes size by IGF-1 pathway activation, suggesting that BET might represent a possible new drug/integrator strategy, not only in sport performance but also in clinical conditions characterized by muscle function impairment.

## Background

Betaine (BET) is a zwitterionic quaternary ammonium compound and it was first discovered in the juice of sugar beets (*Beta vulgaris*) by the German chemist Scheibler, in the 19th century. Now, BET is isolated widely from microorganisms, plants and animals; it is an important component of many foods, including wheat, shellfish and spinach [1].

BET has two main physiologic roles [2]: 1) it is an os-molyte, which accumulated in tissues to regulate cell volume and maintain integrity under hyperosmolar stress [3,4]; 2) it is a methyl donor participating to the methionine-homocysteine cycle. The conversion of homocysteine to methionine is essential to maintain constant methionine level, detoxify homocysteine and produce the universal methyl donor *S*-adenosylmethionine (SAM) [5]. Altered concentration of SAM may influence DNA methylation [6]. Many groups [7,8], including ours [9], showed that DNA methylation is relevant in controlling cellular differentiation, in particular in skeletal muscle development.

Some novel studies suggested a possible role of BET on enhancing exercise performance*.* First, it has been hypothesized that BET consumption improves cardiovascular function and thermo regulation while exercising in a hot environment [10]. Furthermore, in humans, latest evidence proposed BET as a potential ergogenic aid, enhancing strength and power performance probably acting by means of enhancing an increase in skeletal muscle creatine content [11-14]. Overall, previous reports suggest that BET supplementation reduces fatigue and improves muscle function.

Muscle remodeling is a crucial aspect of sport performance. In fact, skeletal muscle is a highly adaptable tissue that is capable not only to increase its mass in response to exercise (hypertrophy), but also to form new fibrils (regeneration) after damage [15-17]. Both muscle processes, hypertrophy and regeneration, are mediated by resident muscle precursor cells, termed satellite cells [18]. Satellite cells are mitotically quiescent. Upon a growth stimulus or injury, satellite cells start proliferating and turn in committed myogenic cells (myoblasts). Myoblasts withdraw from the cell cycle and differentiate first; subsequently elongating and fusing to repair existing damaged myofibers or form new myofibers [19,20]. The sequence of myogenesis depends on highly regulated changes in gene expression, which are coordinated by the myogenic regulatory factors (MRFs) [21]. In particular, the MRFs MyoD and Myf5 act early in myogenesis to determine myogenic fate and to regulate proliferation; Myogenin (Myog) and Myf6 act at later stages of myogenesis to control fusion of myoblasts [22,23]. Other, non-muscle specific transcription factors, such as p21 and MEF2, are also important at specific steps of myogenesis [24,25]. All those regulatory factors coordinate induction of transcription structural muscle-specific genes, such as Myosin Heavy Chain (MyHC, the major structural protein in myotubes). MRFs and several structural proteins extensively regulate cytoskeletal reorganization, which occurs before and after fusion of myoblast [26]. Several reports indicated N-cadherin (N-cad), a member of calcium-dependent cell adhesion molecules, and α sarcomeric actinin (α act), an actin binding protein, having a central role in defining myotubes cytoskeletal architecture [27,28]. Among a variety of extracellular and intracellular molecules, Insulin Growth Factor 1 (IGF-1) promotes the chemiotaxis of satellite cells. The activation of IGF-1 pathway induces both differentiation and hypertrophy of myoblasts [29,30]. IGF-1 actions, including the hypertrophic processes, are mediated by Akt, a serine/threonine kinase, which is a downstream target of IGF-1 signaling [31-33].

The purpose of the present study has assess BET effects during the differentiation and hypertrophic process using C2C12 murine myoblasts. This cell line, derived from satellite cells, differentiate in myotubes after serum removal and provide a useful experimental *in vitro* model to study myogenesis and regulation of skeletal mass [34]. Our results indicate that BET promotes muscle fibers differentiation and myotube hypertrophy via activation of the IGF-1 signaling pathway.

## Methods

### Materials

Anti calnexin (H-70), anti MyoD (C-20), anti Myf5 (C-20), anti Myogenin (D-10), anti Myf6 (C-19), anti IGF-1 (G-17), anti MyHC (H-300), anti N-cadherin (H-63), anti α-tubulin (TU-02), anti p21 (C-19), anti IGF-1 receptor β (C-20), anti Akt (C-20), monoclonal or polyclonal primary antibodies, HRP-conjugated and rhodamine-conjugated secondary antibodies were purchased from Santa Cruz Biotechnology (Santa Cruz, CA, U.S.A.). Anti α sarcomeric actinin primary antibody (A7811) and all other reagents were purchased from Sigma Chem. Co. (St. Louis, MO, U.S.A.). In particular, BET used was betaine monohydrate (Sigma Chem. Co., St. Louis MO, U.S.A.) and according to the manufacturer’s instruction was dissolved in sterile water. Mouse C2C12 myoblastic cells were purchased from the European Collection of Animal Cell Cultures (ECACC).

### Experimental protocol

C2C12 myoblasts, seeded at 6×10^2^ cells/cm^2^, were cultured at 37°C in humidified 5% CO_2_ atmosphere in a growth medium (GM), containing DMEM supplemented with 20% (v/v) FBS (fetal bovine serum), 1% penicillin–streptomycin and 1% L-glutamine. Cell differentiation was initiated by placing 80% confluent cell cultures in differentiation medium (DM), containing DMEM supplemented with 1% HS (horse serum), antibiotics and 1% L-glutamine [23].

In our *in vitro* differentiation model, early myotubes appeared 48 hours (h) after serum starvation (intermediate differentiation phase) and neo myotubes formation was completed after 72 h-96 h (late differentiation phase).

### Dose–response study

To study the dose–response relationship of BET stimulus, we treated neo myotubes with 1, 5 or 10 mM BET for 30 min, 4, 8 and 24 hours (h). Unstimulated neo myotubes represented control cells.

### Myoblasts proliferation and differentiation studies

Based on dose–response results, 10 mM BET concentration was chosen for C2C12 stimulation. To study C2C12 proliferation and differentiation, myoblasts were grown to 40-50% confluence in GM and then stimulated with BET 10 mM. Control cells were maintained in GM or DM. Medium was daily changed and cells inspected for morphological changes.

### Growth curve

C2C12 myoblasts were plated in 35 mm^2^ culture dishes and grown in presence of GM supplement with 10 mM BET. Control cells were cultured in GM. Experiment continued until the control cells have reached sub-confluence (4 days). Medium was daily changed, the cells were counted using a hemocytometer and the average values for each single day were used to plot a growth curve for BET treated and control myoblasts.

### RT^2^-PCR array analysis

We used Mouse Insulin RT^2^-PCR Array plates (PAMM-030A, SABiosciences Corporation, Frederick, MD 21703 USA) to study the BET stimuli dose–response. These plates allow assessing the gene expression involved in IGF-1 pathway, glucose transport and metabolism. To analyze the BET action on cell cycle during myoblasts proliferation, we used Mouse cell cycle RT^2^-PCR Array plates (PAMM-020A, SABiosciences Corporation, Frederick, MD 21703 USA). These plates allow to determinate the expression of 84 genes, among which genes encoding cyclins, positive and negative regulators of cell cycle, as p21 protein. In brief, total RNA was isolated from C2C12 cells using the RNeasy Plus Mini Quiagen kit (Quiagen GmbH, Germany) according to the manufacturer’s instructions. Total RNA (1 μg) was reverse transcribed using RT^2^-First Strand Kit (SABiosciences Corporation, Frederick, MD 21703 USA). The reverse transcripts were used as templates for analysis of gene expression level, using RT^2^-PCR arrays plates, according to the manufacturer’s instructions. Each sample was run in triplicate. We calculated the expression level of the housekeeping genes, chosen for normalization in the threshold cycle (C_t_), and then the fold-change (ΔΔC_t_) for each gene from treatment group compared to the control group. If the ΔΔC_t_ is greater than 1, the result may be reported as a fold up-regulation. If the ΔΔC_t_ is less than 1, the result may be reported as a fold down-regulation.

### Immunoblotting analysis

C2C12 cells were homogenized in lysis buffer (50 mM Tris/HCl, pH 7.4, 150 mM NaCl, 1% Triton X-100, 1 mM sodium orthovanadate (Na_3_VO_4_), 1 mM EDTA, 1 mM PMSF, 1 mg/ml aprotinin, 1 mg/ml leupeptin, 1 mg/ml pepstatin) and shaked for 1 h at 4°C. Detergent-insoluble material was removed from the cell suspension by centrifugation at 12,000 x g for 30 min. Protein contents were quantified using Bradford method. Aliquots of 30 μg supernatant proteins from the different samples were resolved by SDS-PAGE. Electrophoresed proteins were transferred to nitrocellulose membrane (Protran®, Whatman® Schleicher & Schuell) as described previously [35]. The membranes were incubated with specific primary antibodies and then with HRP-conjugated anti species-specific secondary antibodies. To confirm equal protein loading per sample, we used antibody anti calnexin or anti α-tubulin. Immunoreactive bands were visualized by an enhanced chemiluminescence method (Amersham Pharmacia Biotech, Piscataway, NJ, USA). Quantitative measurement of immunoreactive bands was performed by densitometric analysis using the Scion Image software (Scion Corporation, Frederick, MD, USA). The data were then converted to fold change (FC) of the control.

### Immunofluorescence analysis and phase contrast microscopy

For indirect immunofluorescence, cells were fixed in 4% paraformaldehyde, permeabilized with 0.2% Triton X-100, and blocked with PBS containing 1% bovine serum albumin. Cells were then immunostained with specific primary antibody and then with rhodamine-conjugated secondary antibody. Cells were observed using fluorescence microscopy (Leica DM IRE2) and images were captured using IM50 software (Leica Microsystems, Switzerland). To verify that cells number in all conditions was superimposable, nuclei were revealed with DAPI staining. To determinate myotube length dimension, the average measurement on each slide was generated from approximately 150 MyHC positive multinucleated myotubes (at least 3 nuclei). In particular, 10 fields were randomly chosen and all MyHC-positive myotubes were measured. The data were then converted to fold change (FC) of the control. Live C2C12 cells were examined and images acquired by phase contrast microscopy using the same microscope and digital system described above.

### Statistical analysis

Data are presented as the mean ± SD. Statistical significances were calculated using unpaired t-tests. Results were considered significant when p ≤ 0.05.

## Results

### BET dose–response study

**Figure 1:**
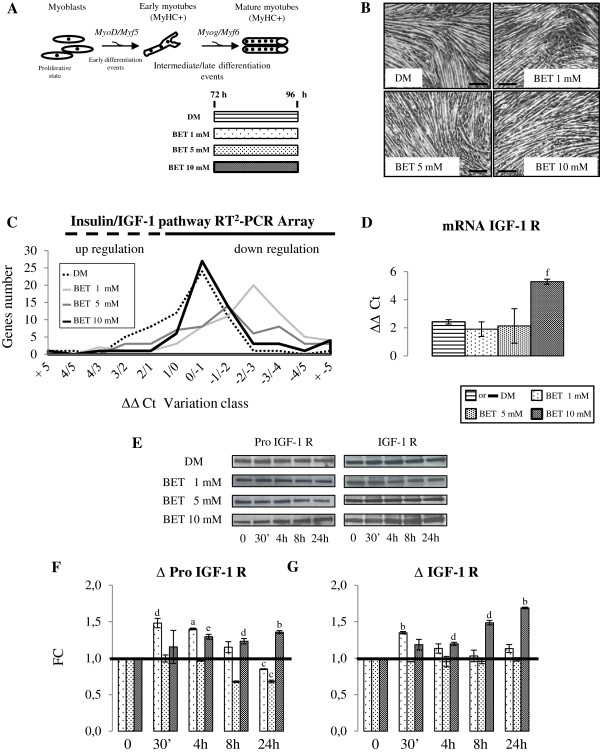
**BET dose–response study. A**. Graphical representation of myogenesis and simplified design of experimental procedures: neo myotubes were treated for 24 h with different concentrations of BET: 1 mM, 5 mM and 10 mM. **B**. Images obtained by brightfield microscopy at the end of experiment (96 h). **C**. Insulin-IGF-1 gene expression profiles after 24 h of BET stimuli. **D**. Analysis of IGF-1 R mRNA: 10 mM BET significantly increased IGF-1 R mRNA content. **E**. Immunoblots representative bands of Pro IGF-1 R and IGF-1 R proteins. **F**.**-G**. Western blot analysis revealed that 10 mM BET improved showing Pro IGF-1 R and IGF-1 R protein levels. We performed three independent experiments. For gene expression analysis data as presented in as fold-change (ΔΔCt) of threshold cycle (Ct) mean ± SD. For immunoblots studies, results are expressed fold changes (FC) mean ± SD. Significance: a = p ≤ 0.05, b = p ≤ 0.04, c = p ≤ 0.03, d = p ≤ 0.02, e = p ≤ 0.01 and f = p ≤ 0.004. Scale bar: 200 μm.

This is the first study to asses BET action on muscle fibers *in vitro*. Therefore a BET dose–response experiment on neo myotubes was performed. C2C12 cells, differentiated for 72 h, were treated for additional 24 h with three different BET doses: 1 mM, 5 mM and 10 mM (as shown in the experimental protocol: Figure 1A).

**Figure 2:**
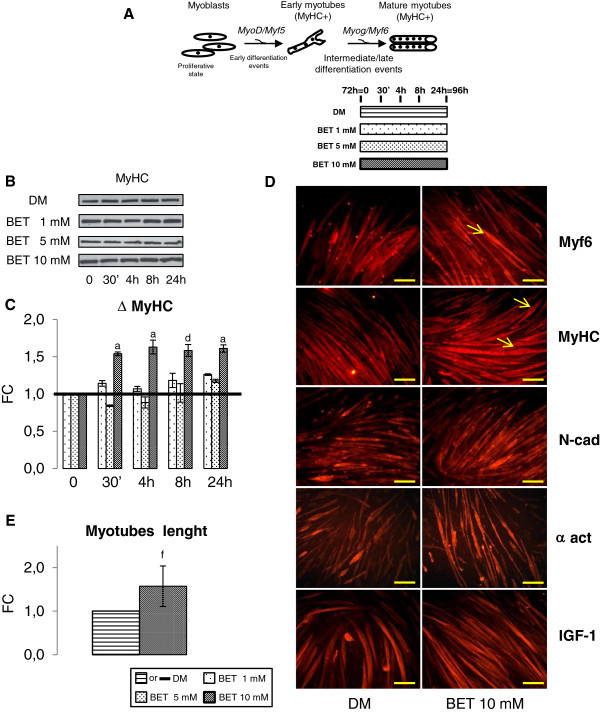
**BET action on neo myotubes features. A**. Graphical representation of myogenesis and **s**implified design of experimental procedures: neo myotubes were treated for 30 min, 4 h, 8 h and 24 h with different concentrations of BET (1 mM, 5 mM and 10 mM). **B**. Representative blot of MyHC protein expression at the indicated times. **C**. Western blot analysis: 10 mM BET raised MyHC protein levels, 1 mM and 5 mM BET did not change MyHC content. **D**. Immunofluorescence analysis of Myf6 and MyHC shows that 10 mM BET stimulated myobutes are characterized by nuclei organization to form a ring (yellow indicator). The same morphological features are evident in N-cad, α act and IGF-1 immunofluorescence studies. **E**. Myotubes length determination: BET myotubes are significant longer than DM myotubes. Data, obtained from three independent experiments, are expressed as fold changes (FC) mean ± SD. Significance: a = p ≤ 0.05, b = p ≤ 0.04, c = p ≤ 0.03, d = p ≤ 0.02, e = p ≤ 0.01 and f = p ≤ 0.004. Scale bar: 200 μm.

Brightfield microscopy revealed that only at the 10 mM BET myotubes length increased (Figure 1B). In Figure 2E, quantitative data confirmed this initial qualitative observation.

Since insulin/IGF-1 signaling is central in skeletal muscle hypertrophic process [29,30], we investigated the molecular mechanisms of BET effect via which BET carries out its action.

Using Mouse Insulin Pathway RT^2^-PCR Array, the gene expression profiles of C2C12 cells stimulated (24 h) with different BET concentrations (1, 5 and 10 mM) and unstimulated cells were compared (Figure 1C). Data obtained confirmed that 1 mM and 5 mM BET did not stimulate the insulin/IGF-1 genes expression network, but 10 mM BET treatment significantly improved IGF-1 R mRNA level (Figure 1D, p ≤ 0.004), without any cytotoxic effect.

Those data suggested that 10 mM BET was able to stimulate cells, activating the IGF-1 R signaling pathway and subsequential improving myotubes length, without causing cellular adverse events.

To confirm this hypothesis, we performed Western blot analysis: neo myotubes were treated with the three different BET concentrations for 30 min, 4 h, 8 h and 24 h (Figure 1E). IGF-1 R is synthesized as a single polypeptide chain (Pro IGF-1 R) that is processed to mature receptor (IGF-1 R) [33]. As shown in Figure 1F, 1 mM BET significantly increased Pro IGF-1 R protein content within 30 minutes and 4 h of treatment (p ≤ 0.02; 4 h: p ≤ 0.05), while Pro IGF-1 R amount notably decreased (p ≤ 0.03) at the end of the experiment, compared to the control cells. We observed a similar reduction of Pro IGF-1 R in neo myotubes stimulated with 5 mM BET for 24 h (p ≤ 0.03), while 5 mM BET did not modify Pro IGF-1 R protein amount at 30 min, 4 h and 8 h. In contrast, 10 mM BET caused a marked increase of Pro IGF-1 R concentration after 4 h up to the end of the study (4-8-24 h: p ≤ 0.01, p ≤ 0.02, p ≤ 0.04). IGF-1 R protein content analysis confirmed the results (Figure 1G): 1 mM BET significantly increased IGF-1 R protein at 30 min only (p ≤ 0.04); 5 mM BET did not determinate any difference. In contrast, 10 mM BET considerably increased IGF-1 R protein level during all time-points of the experiment, with the notable exception of 30 min time-point (4-8-24 h: p ≤ 0.02, p ≤ 0.02, p ≤ 0.04). Those data imply that 10 mM BET action persists beyond 24 h.

### BET action on neo myotubes features

To study BET effect on C2C12 neo myotubes morphology, cells were stimulated for 30 min, 4 h, 8 h and 24 h, using the three different BET concentrations (Figure 2A). MyHC protein content was analyzed by Western blot (Figure 2B). As expected, myotubes treated with 10 mM BET showed a significant MyHC increase (24-48-72-96 h: p ≤ 0.05, p ≤ 0.05, p ≤ 0.02 and p ≤ 0.05). In contrast MyHC protein levels in myotubes supplemented with 1 mM or 5 mM BET only did not show statistical difference compared to blank (Figure 2C).

To verify the hypothesis that 10 mM BET could influence late phase of differentiation program and in particular hypertrophic process, we studied neo myotubes size by immunofluorescence analysis (Figure 2D). Neo myotubes were treated with 10 mM BET and immunostained. Using antibodies against Myf6 and MyHC, a significant increase of number and length of myotubes was detected after stimulation with 10 mM BET compared to DM cells. Furthermore, the images revealed that 10 mM BET treated myotubes are characterized by a particular arrangement of the nuclei to form a ring pattern, which represents a morphological marker of *in vitro* muscle hypertrophy and maturation [36]. Those observations indicate that 10 mM BET is capable to enhance myotubes complete formation. To further prove this hypothesis, we carried out additional immunofluorescence experiments using antibodies against N-cadherin and α sarcomeric actinin. These are skeletal muscle proteins, which play a central role in cytoskeleton rearrangement during myotubes fusion and hypertrophy [27,28]. Immunofluorescence data of N-cadherin and α sarcomeric actinin (Figure 2D) were superimposable to Myf6 and MyHC images, suggesting that 10 mM BET myotubes are more numerous and longer compared with control myotubes. In addition, the length of myotubes treated with BET 10 mM was significantly greater than the control (Figure 2E, p ≤ 0.004). Finally, we performed immunofluorescence assay using antibody against IGF-1 protein. In neo myotubes treated with 10 mM BET the number of IGF-1 positive myotubes was higher than control experiment (Figure 2D).

### BET action on myoblasts proliferation

The start of differentiation is closely related to the regulation of cell cycle. Myoblasts in the G_1_ phase may have three distinct fates: proliferation, commitment to differentiation or entrance into quiescence [37,38].

**Figure 3:**
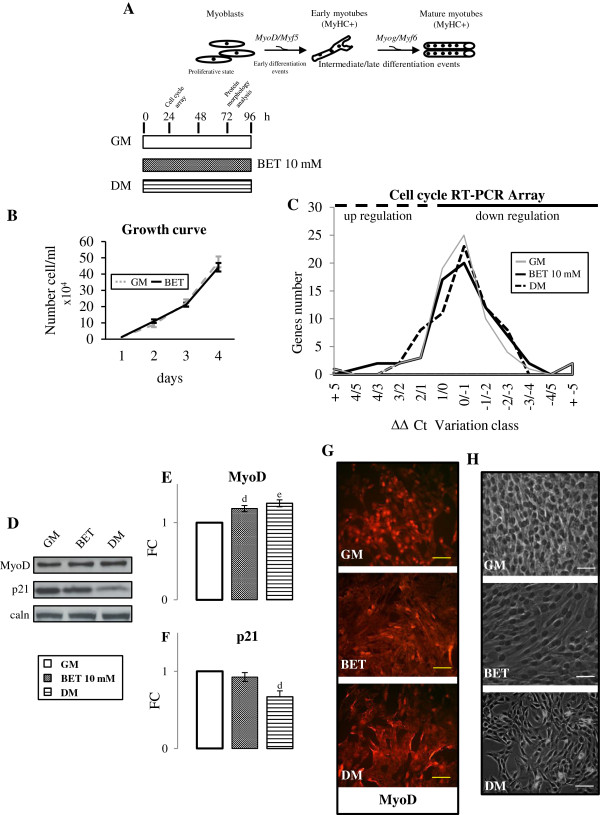
**BET action on proliferative myoblasts. A**. Simplified design of experimental procedures: myoblasts were grown until 40% confluence in GM and then the medium was switched in GM with 10 mM BET or in DM. The experiment continued until the cells reached subconfluence. **B**. Growth curve trend: 10 mM BET did not influence C2C12 proliferative potential. **C**. Cell cycle gene expression after 24 h of treatment: the gene expression profile in the three experimental conditions was similar. **D**. Representative blot of analyzed proteins are shown. **E**-**F**. Western blot analysis: BET 10 mM significantly improved MyoD amount, but did not modified p21 content. **G**-**H**. MyoD immunofluorescence and phase contrast microscopy images revealed that BET 10 mM myoblasts started to assume an elongated shape, similarly to DM. We performed three independent experiments. For gene expression analysis data as presented in as fold-change (ΔΔCt) of threshold cycle (Ct) mean ± SD. Significance: d = p ≤ 0.02, e = p ≤ 0.01. Scale bar: 200 μm.

We investigated 10 mM BET action on C2C12 proliferation (Figure 3A) determining growth curve trend. As shown in Figure 3B, 10 mM BET did not modify C2C12 proliferative potential. Then, we studied 10 mM BET effects on cell cycle using Mouse Cell Cycle RT^2^-PCR array. This array allows characterization of the expression of genes that regulate, both positively and negatively, the progression of cell cycle, the transitions through the different phases, the DNA replication and the checkpoints. Cells were cultured in three different medium for 24 h: GM, GM supplemented with 10 mM BET and DM. As shown in Figure 3C, 10 mM BET did not significantly modify cell cycle factors expression network compared to control experiment and the gene expression profile in the three experimental conditions was similar.

We also measured MyoD and p21 protein levels. MyoD plays a crucial role in achievement and/or maintenance of myogenic phenotype, while p21 has an important function in irreversible withdrawal from the cell cycle [39]. As shown in Figure 3E, 10 mM BET led to a significant increase in MyoD content, similarly to DM, compared with control (BET 10 mM: p ≤ 0,02, DM p ≤ 0,01). Consistently with above mentioned results of cell cycle genes expression profile, 10 mM BET did not modulate p21 protein amount.

Finally, we studied the morphology of myoblasts at the end of the experiment. Images obtained by immunofluorescence analysis using MyoD antibody and phase contrast (Figure 3G-H) showed that myoblasts incubated with 10 mM BET change their morphological aspects: they loose their characteristic flattened morphology, start to become polarized and acquired an elongated form. The morphological changes induced by BET treatment were comparable to those observed in DM condition.

The present data suggest that BET supplement have a minimal effect in promoting myogenic acquisition during proliferative phase. In contrast, BET-mediated morphological changes, associated with MyoD protein level increase, are consistent with the effect of the nutrient in promoting the myoblast commitment to myotube.

### BET action on differentiating myoblasts

**Figure 4:**
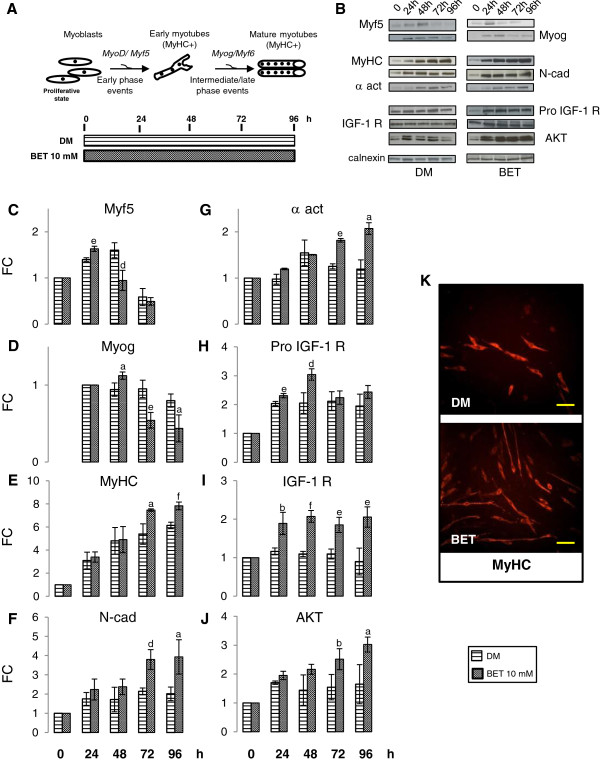
**BET action on differentiating myoblasts. A**. Graphical representation of myogenesis and simplified design of experimental procedures: myoblasts were differentiated in presence of 10 mM BET, differentiation progression was evaluated at early, intermediate and late phase. **B**. Representative bands of analyzed proteins by Western blot are shown. **C**-**D**. Myf5 and Myog immunoblots indicated that BET could regulate MRFs kinetics synthesis, accelerating differentiation progression. **E**-**F**-**G**. Western blot analysis showed that BET significantly enhanced MyHC, N-cad and α act protein content at 72 and 96 h. **H**-**I**-**J**. Western blot studies revealed that, in respect to control, BET 10 mM improved Pro IGF-1 R levels at 24**-**48 h, IGF-1 R amount during all phase of differentiation and AKT levels at 72**-**96 h. **K**. MyHC immunofluorescence analysis at 48 h indicated that BET could influence neo myotubes features, promoting the acquisition of elongated morphology. Data, obtained from three independent experiments, are expressed as fold changes (FC) mean ± SD. Significance: a = p ≤ 0.05, b = p ≤ 0.04, c = p ≤ 0.03, d = p ≤ 0.02, e = p ≤ 0.01 and f = p ≤ 0.003. Scale bar: 200 μm.

We investigated BET effect on the several phases of myogenesis (Figure 4A). Sequential expression of MRFs is important for the successful of myogenesis program [16,19,21]. In particular, Myf5 is required for commitment to the myogenic lineage and it is mainly expressed during early phase, while Myog drives the early of myotubes genesis and its expression is limited to the intermediate phase (48 h). To study myogenesis progression in presence of 10 mM BET, the protein levels of Myf5 and Myog were measured. As shown in Figure 4C, Western blot analysis indicated that, in presence of BET, Myf5 maximum increment was observed 24 h after the induction of differentiation with respect to the control (p ≤ 0.01). In control condition, Myf5 activation peak was observed at 48 h. In contrast, in BET treated myoblasts, Myf5 dramatically decreased at 48 h with respect to the control condition (p ≤ 0.02). Similarly, in BET myoblasts we observed Myog expression peak at 48 h (Figure 4D, p ≤ 0.05), followed by a rapid protein content reduction. In contrast, in control cells Myog protein level remained unchanged up to 72 h. Ours results indicate that BET stimulates the kinetics of Myf5 and Myog synthesis and enhances skeletal muscle cells differentiation process.

To further define this effect, we analyzed MyHC protein level. As shown in Figure 4E, during early and intermediate phases of differentiation (24 and 48 h, respectively) we did not find a significant difference between MyHC protein levels in BET treated cells with respect to control cells. In contrast, we detected a considerable increase of MyHC protein amount in 10 mM BET cells during late and terminal phases of differentiation with respect to the control cells (72 h-96 h: p ≤ 0.05, p ≤ 0.003).

During late and terminal differentiation phases, fusion of myoblast in new myotubes is a central event. Fusion is a complex mechanism and requires cytoskeletal rearrangement [26,36]. Thus, we determinated N-cad protein content during differentiation. As shown in Figure 4F, 10 mM BET markedly increased N-cad protein level at the end of differentiation (72 h-96 h: p ≤ 0.02, p ≤ 0.05 with respect to the control). Furthermore, we observed the same results when we analyzed α sarcomeric actinin protein level (72 h-96 h: p ≤ 0.01, p ≤ 0.05 vs control, Figure 4G). Thus, BET could positively regulate the differentiation process and, in particular, myoblast fusion via an effect on cytoskeleton proteins network.

To confirm this hypothesis, we investigated whether BET was able to modify morphological features of C2C12 cells after 48 h from differentiation induction, when myoblasts start to fuse in new myotubes. Using an antibody against MyHC, we observed that 10 mM BET supplement induced a higher number of new myotubes, which were longer than control (Figure 4K). Thus, BET modulates C2C12 myotubes size promoting cell elongation. The higher number of MyHC positive myotubes in 10 mM BET condition confirmed Western blot data and the hypothesis that BET addition to DM enhances the progression of cell differentiation.

Finally, we evaluated the IGF-1 signaling pathway. As shown in Figure 4H, BET markedly increased Pro IGF-1 R level at 24 and 48 h (p ≤ 0.01, p ≤ 0.02, respectively). During all phases, IGF-R amount was significant higher in BET 10 mM cells compared to control (Figure 4I). Western blot analysis indicated a significant increment of AKT, a key kinase of signaling cascade activated by IGF-1R [31-33] in BET cells after 72 h and 96 h (Figure 4J: p ≤ 0.04, p ≤ 0.05) with respect to control cells.

## Discussion

We presently report a set of experiments studying the role of BET on neo myotubes maturation and differentiation in C2C12 cells.

Recent studies have assessed the potential use of BET as an ergogenic aid in athletic performance [10-14,40,41]. Previous authors have suggested that BET acts as osmoprotector and a methyl donor [10,11,13], although no focused *in vitro* studies were carried out to investigate the cellular and molecular mechanisms of BET on skeletal muscle differentiation and hypertrophy.

At our knowledge, this is the first *in vitro* study analyzing BET effect on the C2C12 cells committment, the differentiation process and the morphology of neo myotubes.

Firstly, we observed that BET enhances neo myotube formation, as indicated the MyoD analysis during proliferation phase (Figure 3G) and the kinetics synthesis of Myf5/Myog during the differentiation phase (Figure 4C-D). The effect of BET on cytoskeleton protein levels (MyHC, N-cad, α sarcomeric actinin) and morphological analysis suggests that BET acts on early stage of hypertrophy, accelerating it.

Secondly, another novel aspect of our work is constituted by the finding that BET supplement activates IGF-1 signaling pathway: this is in accordance with previous work suggesting that BET could modulate IGF-I signaling [42].

Satellite cells have the function of reserve myoblasts and C2C12 cells represent the best immortalized model of them. In response to regular exercise training, skeletal muscle can increase its size and contractile power [16,17,21]. During exercise, satellite cells are activated and fuse in pre-existing myofibers. Projecting our data to the *in vivo* condition, we can speculate that BET, through IGF-1 signaling pathway activation, may cause hypertrophy and eventually ameliorate exercise performance.

Ageing, sedentary lifestyles, immobilization, neuromuscular disorders and chronic degenerative diseases (such as atherosclerosis, cancer and type 2 diabetes), are associated with loss of skeletal muscle mass and reduced contractive force [17,43,44]. It has been observed that, in response to injury, satellite cells can activate and fuse with damaged myofibers to promote repair and regeneration. Obesity is characterized by a state of chronic low-grade inflammation and of high circulating and tissue inflammatory markers, besides being characterized by relative sarcopenia [44,45]. BET could influence skeletal muscle regeneration in positive manner and inflammation process in negative manner. Recently, Olli et al. demonstrated that extensive changes in the expression of inflammation-related adipokines in human adipocytes, caused by hypoxia, could be diminished by the presence of physiological concentrations of BET [45]. Similarly, obesity and ageing are associated with profound alterations in epigenetic patterns [46,47]. Future investigations might focus on the effect of BET on epigenetic profile and on the regulation of SAM levels.

Several authors suggested that IGF-1 pathway operates in an autocrine/paracrine mode acting as an intrinsic mediator of skeletal muscle repair and adaptation, increasing the proliferation potential of satellite cells, promoting their differentiation, enhancing muscle regeneration and eventually determining protein synthesis and increase muscle mass [29,30,33,37].

Our data indicate BET as a positive stimulus for the activation of IGF-1 pathway in skeletal muscle. In recent years, the potential effects of BET supplementation in content of meat livestock were investigated. When added to animal feeds, BET enhanced lean muscle mass and reduced the fat [48]. During BET treatment, animals showed an increase in muscle mass, but also in growth hormone, IGF-1 and insulin blood concentrations [49-51], providing additional evidence of the relationship between BET action on skeletal muscle and IGF-1 signaling. Most importantly, very recently, Apicella et al., have demonstrated that BET supplementation significantly increased IGF-1, AKT content as well as the respective anabolic signaling environment in skeletal muscle of trained men [52].

## Conclusions

In summary, our *in vitro* work provides the first evidence of possible BET positive action on skeletal muscle myoblasts differentiation, in particular on the progression of the differentiation process and on myotubes morphology. This effect is at least partially mediated by the IGF-1 signaling activation. Our *in vitro* results are consistent with *in vivo* data obtained in livestock and in humans and may contribute the bench proof for a use of BET as a dietary supplement in humans.

## Competing interests

The authors declare that they have no competing interests.

## Authors’ contributions

PS, AM and IT designed and conducted the research and wrote the manuscript; PS, LL, AM, NM and IT analyzed the data. IT had primary responsibility for the final content. All authors read and approved the final manuscript.
